# Retraction: An improved beluga whale optimizer—Derived Adaptive multi-channel DeepLabv3+ for semantic segmentation of aerial images

**DOI:** 10.1371/journal.pone.0320728

**Published:** 2025-03-17

**Authors:** The PLOS One Editors

Following publication of [1], concerns were raised that this article contains a substantial degree of overlap with a previously published article [2] by the same authors with regard to the article text, reported methodology, equations, datasets, and quantitative results.

The corresponding author stated that there are differences in the literature review and that only two performance metrics are analyzed in [1] whereas additional analyses are included in [2], noting that the work in [2] is an extension of [1]. On the basis of PLOS’ editorial assessment and input received from a member of the *PLOS One* Editorial Board, the *PLOS One* Editors consider the extent of overlap is such that this article [1] constitutes a redundant publication.

The *PLOS One* Editors retract this article [1] because, per our editorial assessment, it does not meet *PLOS One*’s publication criteria (#2) [3].

All authors did not agree with the retraction and stand by the article’s findings.

The retracted article [1] was removed from the *PLOS One* website on March 5, 2025, due to the similarity with [2]. The article’s Copyright and Data Availability statements were also updated at that time, and the removed contents are no longer offered under the Creative Commons Attribution License.

## References

[1] P A, P V. An improved beluga whale optimizer-Derived Adaptive multi-channel DeepLabv3+ for semantic segmentation of aerial images. PLoS One. 2023;18(10):e0290624. doi: 10.1371/journal.pone.0290624 37903154 PMC10615319

[2] Anilkumar P, Venugopal P. An adaptive multichannel DeepLabv3 + for semantic segmentation of aerial images using improved Beluga Whale Optimization Algorithm. Multimed Tools Appl. 2023;83(15):46439–78. doi: 10.1007/s11042-023-17247-z

[3] https://journals.plos.org/plosone/s/criteria-for-publication#loc-2

